# Checklist of recent brachiopod species collected during the Terrasses and Exbodi cruises in the New Caledonian region, SW Pacific

**DOI:** 10.3897/zookeys.537.6567

**Published:** 2015-11-18

**Authors:** Maria Aleksandra Bitner

**Affiliations:** 1 Institute of Paleobiology, Polish Academy of Sciences, ul. Twarda 51/55, PL-00-818 Warszawa, Poland

**Keywords:** Brachiopoda, biodiversity, New Caledonia, South-West Pacific

## Abstract

Twenty species belonging to 16 genera, i.e. *Neoancistrocrania*, *Novocrania*, *Basiliola*, *Basiliolella*, *Ebiscothyris*, *Stenosarina*, *Kanakythyris*, *Xenobrochus*, *Terebratulina*, *Eucalathis*, *Fallax*, *Frenulina*, *Septicollarina*, *Campages*, *Annuloplatidia*, and *Thecidellina* have been identified in the material collected during the Terrasses and Exbodi cruises in the New Caledonian region, SW Pacific. The species *Basiliolella grayi* (Woodward, 1855) and *Fallax neocaledonensis* Laurin, 1997 are the most common in the studied collection, while *Eucalathis murrayi* (Davidson, 1878) is reported for the first time from the New Caledonian region.

## Introduction

The New Caledonia Exclusive Economic Zone, comprising the area from Vanuatu to Chesterfield Islands, is one of the most intensively investigated regions in the Indo-West Pacific Province; more than 40 oceanographic expeditions have been organized by French institutions within the programme Tropical Deep-Sea Benthos (formerly Musorstom; see also Bouchet et al. 2008). Brachiopods collected in this region have been described in many publications (e.g. d’Hondt 1987; Laurin 1992, 1997; Bitner 2007a, 2009, 2010, 2011; Bitner et al. 2008; Bitner and Cohen 2015).

**Figure 1.**
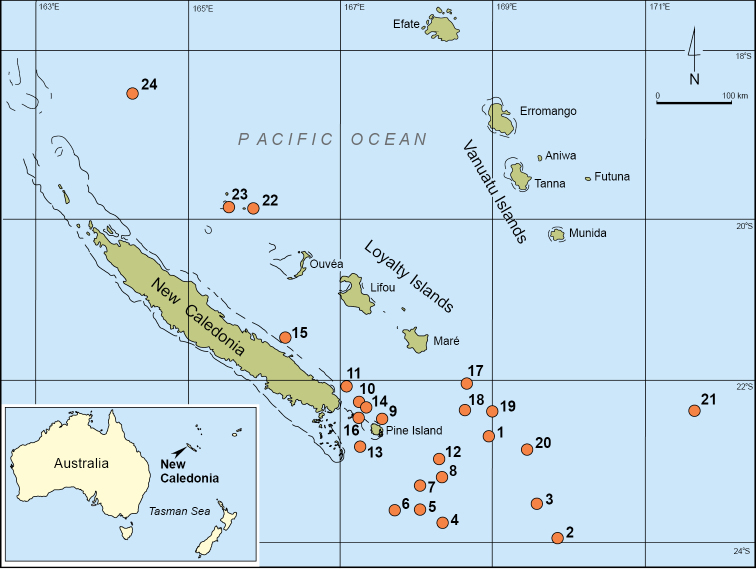
Location map of the brachiopod-bearing stations of the Terrasses and Exbodi expeditions. **1** DW 3032 **2** DW 3039–3042; **3** CP 3047 **4** CP 3051 **5** DW 3056 **6** DW 3059–3060 **7** DW 3062–3063, CP 3065–3068, DW 3069, CP 3070 **8** DW 3072, DW 3075–3077 **9** DW 3078–3079, DW 3082–3083 **10** DW 3086, DW 3089–3090, CP 3091 **11** DW 3093–3094, CP 3834 **12** DW 3100, DW 3102, CP 3104, DW 3106–3110 **13** DW 3120–3124, DW 3129 **14** DW 3784–3785, CP 3786, DW 3787, CP 3788–3789, CP 3791–3793 **15** DW 3798 **16** CP 3842–3844, DW 3845 **17** DW 3846, CP 3848–3849 **18** CP 3851–3852, DW 3896, CP 3898, DW 3900 **19** DW 3862–3863 **20** CP 3871, DW 3872 **21** DW 3880, CP 38882–3885, DW 3887, DW 3889, DW 3895 **22** DW 3902–3903, DW 3913, DW 3916–3918 **23** DW 3905–3907, CP 3911 **24** DW 3922–3925, CP 3927, DW 3928, DW 3930, DW 3932–3933, DW 3949–3940.

This paper deals with brachiopods collected during two cruises, Terrasses and Exbodi, organized by the Muséum national d’Histoire naturelle, Paris and by the Institut de la Recherche pour le Développement, Nouméa, New Caledonia on R.V. “*Alis*” (Fig. 1). The cruise Terrasses (http://expeditions.mnhn.fr/campaign/terrasses) was carried out from 15 to 31 October 2008, south of New Caledonia, whereas the cruise Exbodi (http://expeditions.mnhn.fr/campaign/exbodi) to the Loyalty Ridge, east of New Caledonia was carried out from 2 to 28 September 2011. Samples were collected using a Warén dredge (DW) or a trawl (CP, CC). The brachiopods were found in 46 of 99 Terrasses stations, and in 56 of 161 Exbodi stations. See the Appendix for details of the stations and species distributions. The collections are stored in the Muséum national d’Histoire naturelle, Paris under catalogue numbers IB-2013-171 to IB-2013-271, IB-2013-516 to IB-2013-552, and IB-2013-585 to IB-2013-616.

## Results

The brachiopod fauna recognized in the Terrasses and Exbodi cruises consists of 20 species belonging to 16 genera in 11 families (Craniidae, Basiliolidae, Terebratulidae, Dyscoliidae, Cancellothyrididae, Chlidonophoridae, Aulacothyropsidae, Frenulinidae, Dallinidae, Platidiidae, Thecidellinidae), four orders (Craniida, Rhynchonellida, Terebratulida, Thecideida), and two subphyla (Craniiformea, Rhynchonelliformea).

### Family Craniidae Menke, 1828

***Neoancistrocrania norfolki* Laurin, 1992**

Fig. 2A–B

This species, represented only by young individuals, was found only in two Exbodi stations at depths of 388–520 m. *Neoancistrocrania norfolki* differs from other craniids by its massive ventral valve and internally by two erect divergent processes on the dorsal valve (Laurin 1997; Bitner 2009). Originally described from the Norfolk Ridge (Laurin 1992), it seems to be restricted to the Western Pacific (Cohen et al. 2014).

**Figure 2.**
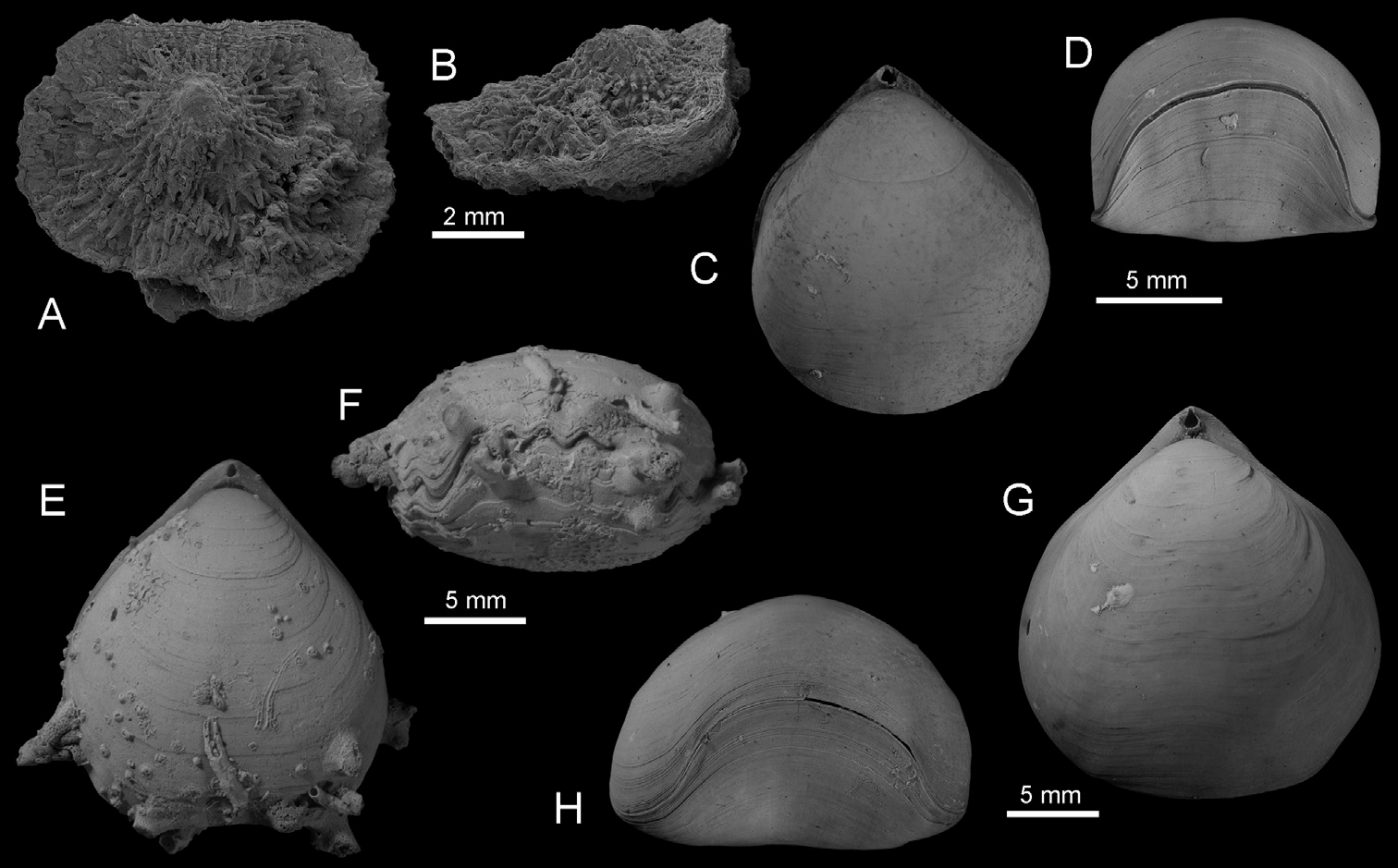
**A–B** *Neoancistrocrania norfolki* Laurin, 1992, dorsal and lateral views of complete specimen (IB-2013–600), SEM, cruise Exbodi, stn DW 3925, 388 m **C–D** *Basiliola lucida* (Gould, 1862), dorsal and anterior views of complete specimen (IB-2013–542), cruise Exbodi, stn DW 3900, 366–357 m **E–F** *Basiliolella grayi* (Woodward, 1855), dorsal and anterior views of complete specimen (IB-2013–188), cruise Terrasses, stn DW 3062, 300–320 m **G–H** *Basiliola beecheri* (Dall, 1895), dorsal and anterior views of complete specimen (IB-2013–215), cruise Terrasses, stn DW 3083, 470–570 m.

***Novocrania* sp.**

The second craniid brachiopod is very rare and too poorly preserved to permit identification to species level. It was found in two stations (600–802 m). Morpho-species identification of *Novocrania* is uncertain and previous specimens from the region of New Caledonia have been described as *Novocrania reevei* Lee & Brunton, 1986 (see Bitner 2010). In the opinion of Robinson and Lee (2011) this is a synonym of *Novocrania japonica* (Adams, 1863).

### Family Basiliolidae Cooper, 1959

***Basiliola beecheri* (Dall, 1895)**

Fig. 2G–H

This is one of three rhynchonellide species recognized in the studied material. *Basiliola beecheri* is relatively common, found in 10 samples, with a depth range of 400–990 m and was already reported from the New Caledonian region (Laurin 1997; Zezina 2005; Bitner 2009). It also occurs in the Hawaii and Fiji regions (Dall 1895; Bitner 2006b, 2008).

***Basiliola lucida* (Gould, 1862)**

Fig. 2C–D

This species, already noted from New Caledonia (Laurin 1997; Bitner 2009), is rare, being found in 5 stations. Its depth range in the studied area is 300–510 m. Originally described from off Japan (Hatai 1940), *Basiliola lucida* also occurs in the Fiji region (Bitner 2008).

***Basiliolella grayi* (Woodward, 1855)**

Fig. 2E–F

This is one of the most common species (nearly 500 specimens). It was found in 13 Terrasses stations and 12 Exbodi stations (see Appendix), with a depth range of 150–584 m. *Basiliolella grayi* is restricted to the SW Pacific (Laurin 1997; Logan 2007; Bitner 2009).

### Family Terebratulidae Gray, 1840

***Ebiscothyris bellonensis* Bitner & Cohen, 2015**

Fig. 3E–J

This species, recently described from the Coral Sea by Bitner and Cohen (2015), is common only in the material collected during the Exbodi cruise. Its depth range is very great, from 70 to 1180 m. Externally, *Ebiscothyris bellonensis* is very similar to *Abyssothyris wyvillei* (Davidson, 1878), but it differs internally in the character of the loop; in *Abyssothyris wyvillei* the loop has a narrow, anteriorly convex transverse band (see Cooper 1983; Bitner 2006b, 2008), while in *Ebiscothyris bellonensis* the transverse band is broad and medially folded. Molecular analysis confirms this separation (Bitner and Cohen 2015).

**Figure 3.**
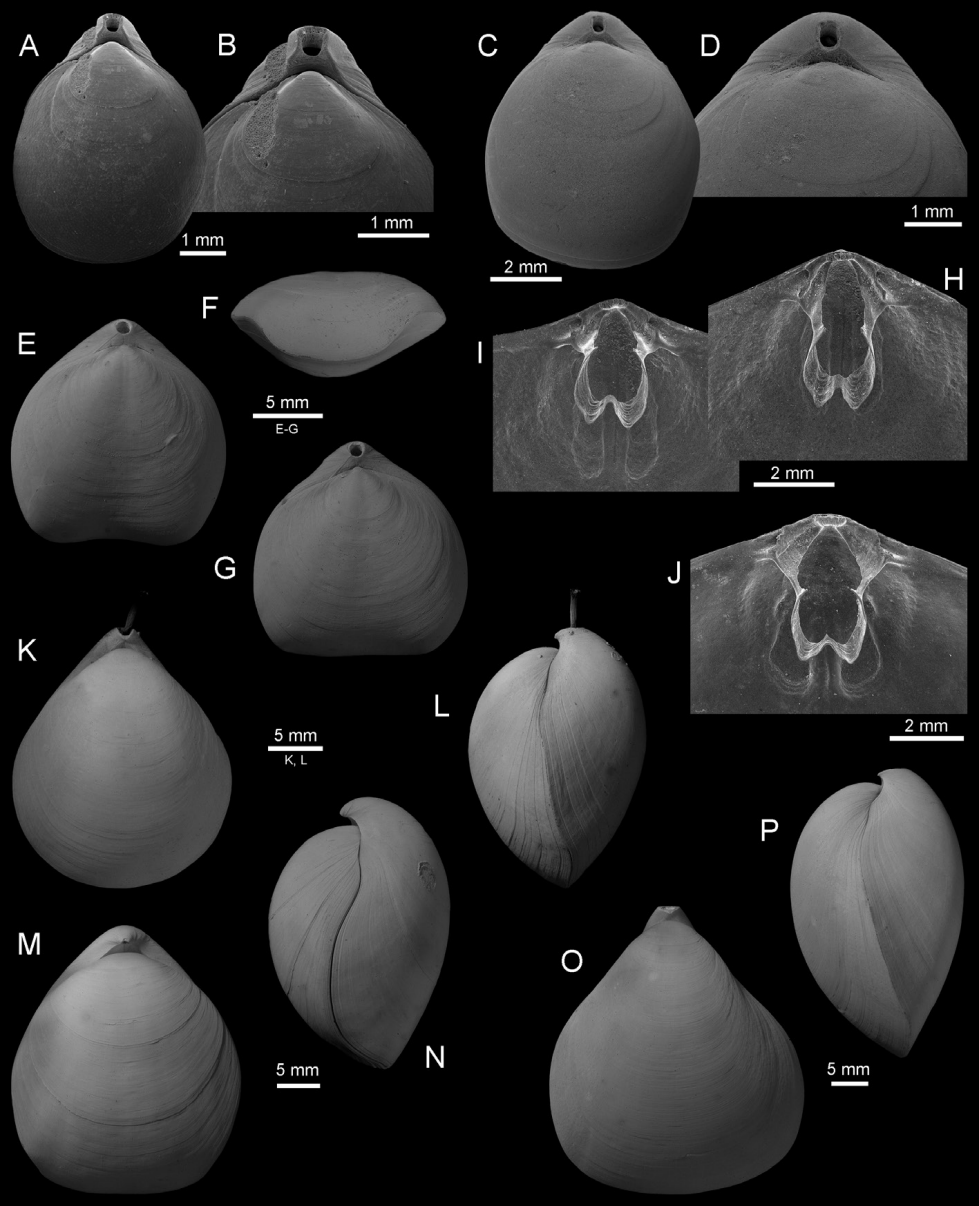
**A–B** *Xenobrochus africanus* (Cooper, 1973), dorsal view of complete specimen (IB-2013–236), and enlargement of the posterior part to show details of the beak, SEM, cruise Terrasses, stn DW 3109, 150–180 m **C–D** *Xenobrochus indianensis* (Cooper, 1973), dorsal view of complete specimen (IB-2013–602), and enlargement of the umbonal part to show details of the beak, SEM, cruise Exbodi, stn DW 3925, 388 m **E–J** *Ebiscothyris bellonensis* Bitner & Cohen, 2015, cruise Exbodi, **E–G** dorsal and anterior views of complete specimens (IB-2013–262), stn CP 3844, 815–970 m **H–I** interior and tilted (**I**) views of dorsal valve (IB-2013–262), SEM, stn CP 3844 **J** interior of dorsal valve (IB-2013–254), SEM, stn CP 3791, 750–863 m **K–L** *Stenosarina globosa* Laurin, 1997, dorsal and lateral views of complete specimen (IB-2013–227), cruise Terrasses, stn DW 3102, 410–430 m **M–N** *Kanakythyris pachyrhynchos* Laurin, 1997, dorsal and lateral views of complete specimen (IB-2013–231), cruise Terrasses, stn DW 3107, 380–440 m **O–P** *Stenosarina crosnieri* (Cooper, 1983), dorsal and lateral views of complete specimen (IB-2013–175), cruise Terrasses, stn DW 3041, 800–840 m.

***Stenosarina crosnieri* (Cooper, 1983)**

Fig. 3O–P

This short-looped terebratulide is a relatively common species in the investigated material, being already recorded from New Caledonia (Laurin 1997; Bitner 2009). About 50 specimens were found in 17 stations, with a depth range of 340–951 m. *Stenosarina crosnieri* was originally described from the south-western Indian Ocean (Cooper 1983).

***Stenosarina globosa* Laurin, 1997**

Fig. 3K–L

The second *Stenosarina* species in the studied material is much rarer, being found in only five samples (386–570 m). *Stenosarina globosa* is smaller than *Stenosarina crosnieri* and characterized by a strongly convex shell. So far known only from the New Caledonia area (Laurin 1997), this species can be considered as endemic to this region.

***Kanakythyris pachyrhynchos* Laurin, 1997**

Fig. 3M–N

This species is very characteristic with its thick shell, strongly incurved beak and very small foramen. It is relatively rare, being found in six samples (150–510 m). It is known only from the New Caledonian region (Laurin 1997; Bitner 2009) and can be regarded as endemic to this area.

### Family Dyscoliidae Fischer & Oehlert, 1890

***Xenobrochus africanus* (Cooper, 1973)**

Fig. 3A–B

A single specimen of this species was found in one Terrasses station at 150–180 m but it was earlier recorded from New Caledonia (Laurin 1997; Bitner 2010). *Xenobrochus africanus* was originally described from South Africa (Cooper 1973).

***Xenobrochus indianensis* (Cooper, 1973)**

Fig. 3C–D

This second species of *Xenobrochus* is also very rare, found in one Exbodi station at a depth of 388 m. Laurin (1997) already noted this species from New Caledonia. It was originally described from South Africa (Cooper 1973). *Xenobrochus indianensis* can be distinguished from *Xenobrochus africanus* by a more convex shell, incurved beak with a partly concealed symphytium, and internally by the presence of a distinct cardinal process (Cooper 1973, 1983; Laurin 1997).

### Family Cancellothyrididae Thomson, 1926

***Terebratulina pacifica* Yabe & Hatai, 1934**

Fig. 4D

This is a relatively common species, found in 12 stations (180–790 m). Its presence in the studied area was already noted (Laurin 1997; Bitner 2009), and it is common off Japan (Hatai 1940). This wide distribution suggests that a careful molecular analysis of a wide range of Pacific Ocean samples might lead to the recognition of multiple forms that have not been distinguished morphologically (e.g. Lüter and Cohen 2002).

**Figure 4.**
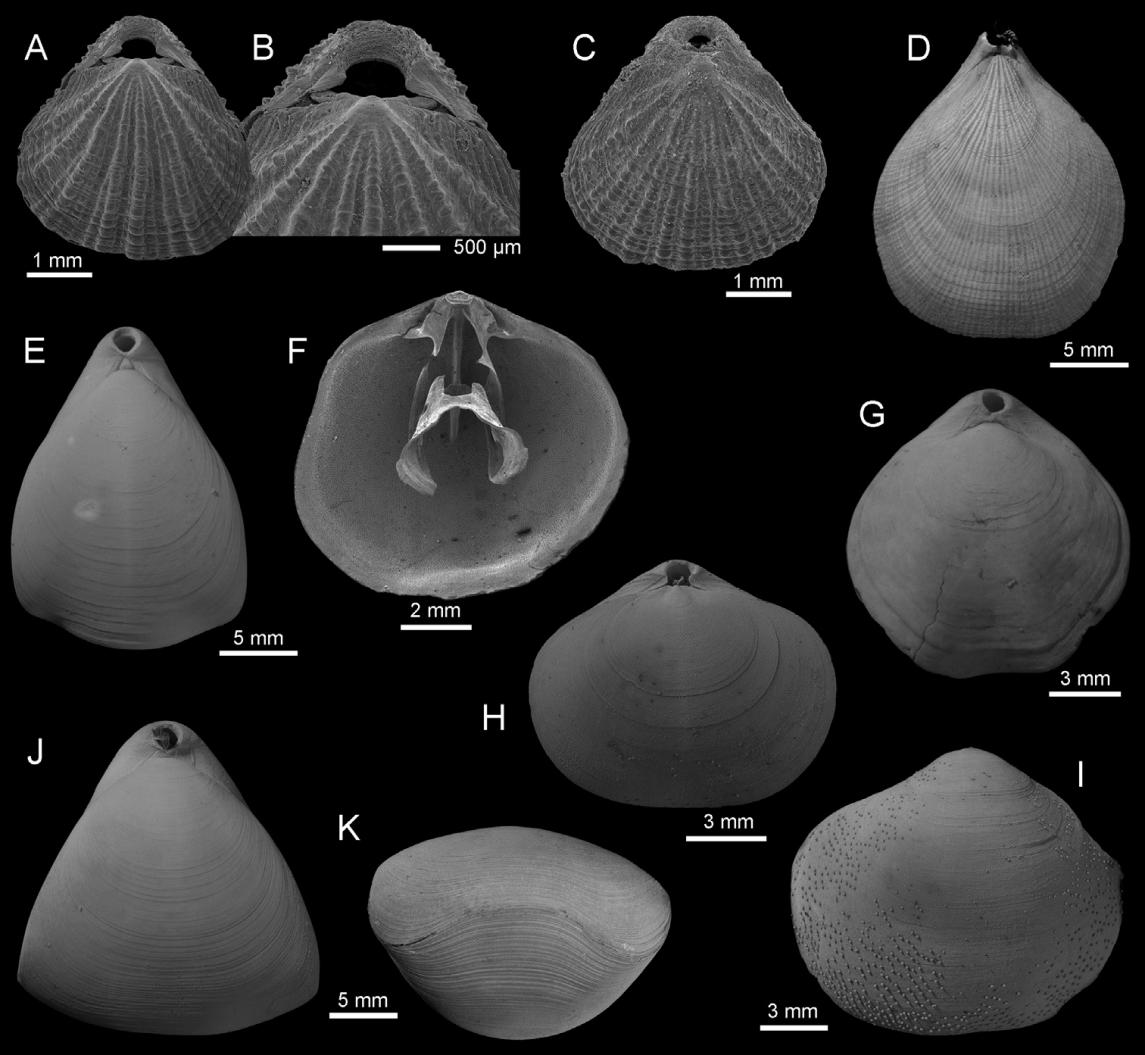
**A–C** *Eucalathis murrayi* (Davidson, 1878), cruise Exbodi, SEM **A–B** dorsal view of complete specimen (IB-2013–601), and enlargement (**B**) of posterior part to show details of the beak, stn DW 3925, 388m **C** dorsal view of complete specimen (IB-2013–588), stn CP 3911, 680–802 m **D** *Terebratulina pacifica* Yabe & Hatai, 1934, dorsal view of complete specimen (IB-2013–214), cruise Terrasses, stn DW 3082, 290 m **E** *Campages mariae* (Adams, 1860), dorsal view of complete specimen (IB-2013–259), cruise Exbodi, stn CP 3834, 27–258 m **F–G** *Frenulina sanguinolenta* (Gmelin, 1791) **F** interior of dorsal valve (IB-2013–525), SEM, cruise Exbodi, stn 3872, 159–756 m **G** dorsal view of complete specimen (IB-2013–192), cruise Terrasses, stn DW 3063, 430–480 m **H–I** *Septicollarina zezinae* Bitner, 2009, cruise Terrasses, stn DW 3040, 750–780 m (IB-2013–173) **H** dorsal view of complete specimen **I** exterior of ventral valve, visible randomly distributed, small pustules **J–K** *Fallax neocaledonensis* Laurin, 1997, dorsal and anterior views of complete specimen (IB-2013–210), cruise Terrasses, stn DW 3077, 420–540 m.

### Family Chlidonophoridae Muir-Wood, 1959

***Eucalathis murrayi* (Davidson, 1878)**

Fig. 4A–C

This species is very rare and was found in only two Exbodi stations (388–802 m). Although known from the nearby New Zealand region (MacFarlan et al. 2009), this is the first report of *Eucalathis murrayi* from the vicinity of New Caledonia. Originally described from off the Kermadec Islands (Davidson 1880) it has a wide distribution in the south-western Pacific (Bitner 2006a) and is known from the western Indian Ocean (Zezina 1987).

So far the only representative of the family Chlidonophoridae reported from New Caledonia has been *Eucalathis rugosa* Cooper, 1973 (see Laurin 1997; Bitner 2009, 2010), which is characterized by strong ornamentation of single, coarse ribs, while in *Eucalathis murrayi* ribs are numerous, finer, and bifurcating. DNA sequences of these species have not yet been compared (B.L. Cohen, pers. communication).

### Family Aulacothyropsidae Dagys, 1972

***Fallax neocaledonensis* Laurin, 1997**

Fig. 4J–K

This long-looped brachiopod is one of the most common (more than 250 specimens) and was identified in 20 stations (260–840 m). *Fallax neocaledonensis* was originally described from New Caledonia by Laurin (1997; see also Bitner 2009) and was also recognized in material from Fiji (Bitner 2006b, 2008).

***Septicollarina zezinae* Bitner, 2009**

Fig. 4H–I

This rare species was found in one station of each cruise (680–802 m). Originally described from the Norfolk Ridge (Bitner 2009) it was also identified around Fiji and French Polynesia (Bitner 2008, 2014).

### Family Frenulinidae Hatai, 1938

***Frenulina sanguinolenta* (Gmelin, 1791)**

Fig. 4F–G

This easily recognizable species was found in 20 stations but was abundant only in the Exbodi material where it appears to have a very wide bathymetric range (110 to 1100 m). However this range probably is the result of the wide depth-range of the DW3932 dredge haul (500 to 1100 m), the specimens of this generally shallow-water form probably having been collected only in the shallower water.

*Frenulina sanguinolenta* is one of the most widely distributed species of living brachiopods, known from Japan, Australia, New Caledonia, Fiji, French Polynesia and Hawaii (Hatai 1940; Emig 1987; Saito 1996; Laurin 1997; Bitner 2006a, 2006b, 2007a, 2008, 2009, 2010, 2014). Recently this species has also been identified in the western Indian Ocean (Bitner and Logan in press).

### Family Dallinidae Beecher, 1893

***Campages mariae* (Adams, 1860)**

Fig. 4E

In the studied material this species was found in 16 stations in the material of both cruises at depths of 180–790 m. Originally described from off Japan (Hatai 1940), *Campages mariae* occurs in the Western Pacific (Logan 2007; Bitner 2009, 2010).

### Family Platidiidae Thomson, 1927

***Annuloplatidia richeri* Bitner, 2009**

Fig. 5F–J

**Figure 5.**
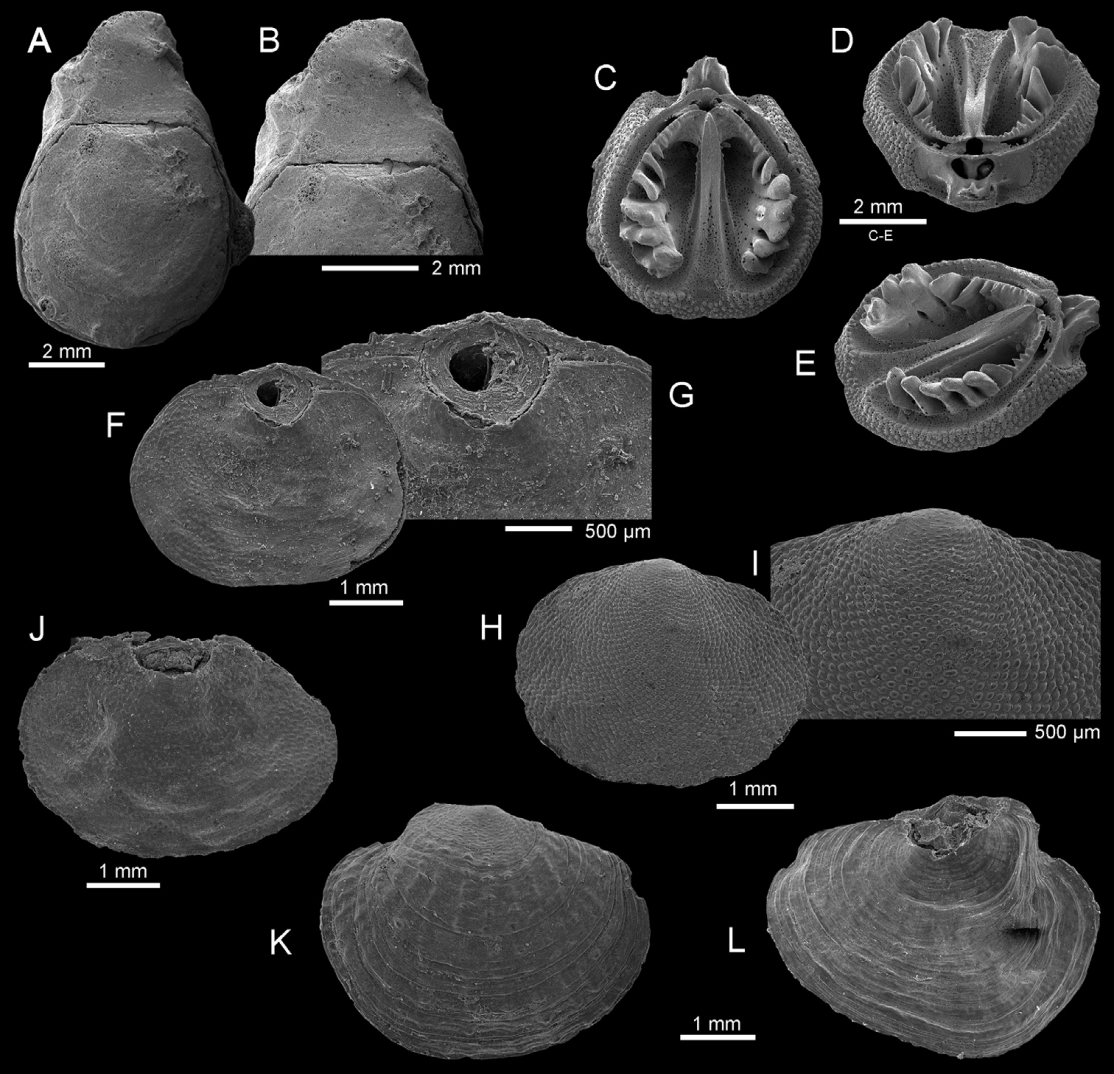
**A–E** *Thecidellina maxilla* (Hedley, 1899), cruise Exbodi, stn DW 3905, 300 m, (IB-2013–549) **A–B** dorsal view of complete specimen and enlargement of the posterior part to show flat pseudodeltidium (planodeltidium) **C–E** inner, posterior (**D**) and oblique (**E**) views of dorsal valve to show bridge, median lobe of cardinal process and median septum **F–J** *Annuloplatidia richeri* Bitner, 2009 **F–G** dorsal view of complete specimen (IB-2013–616), and enlargement of the umbonal part, cruise Terrasses, stn DW 3040, 750–780 m **H–I** ventral view of complete specimen (IB-2013–592), and enlargement of shell surface to show nodes, cruise Exbodi, stn DW 3913, 622 m **J** dorsal view of complete specimen (IB-2013–590), cruise Exbodi, stn DW 3911, 680–802 m **K–L** *Annuloplatidia curiosa* Bitner, 2014, ventral and dorsal views of complete specimen (IB-2013–522), cruise Exbodi, stn DW 3862, 400–520 m. All SEM.

This species was originally described from the Norfolk Ridge where it was very common (see Bitner 2009). Here, *Annuloplatidia richeri* is rare, found in one Terrasses station and two Exbodi stations (622–802 m). It is characterized by numerous, transversely elongate pustules that cover the ventral valve (Fig. 5H–J). *Annuloplatidia richeri* can be treated as endemic to the New Caledonian region as it has not yet been recognized in other areas.

***Annuloplatidia curiosa* Bitner, 2014**

Fig. 5K–L

This species is very rare, found in only one Exbodi station at depths of 400–520 m. It was already reported from New Caledonia by Laurin (1997), but was wrongly assigned by him to *Megerlia echinata* (Fischer & Œhlert, 1890) (see discussion in Bitner 2014: 256). It has a very wide distribution, being known from New Zealand, Wallis and Futuna Islands, and French Polynesia (Bitner 2007b, 2008, 2014).

### Family Thecidellinidae Elliott, 1953

***Thecidellina maxilla* (Hedley, 1899)**

Fig. 5A–E

This is the only thecideide brachiopod in the investigated material. This species was found in 10 Exbodi stations with a very wide depth range (159 to 1100 m) but as noted before (see *Frenulina sanguinolenta* above) this may be an artefact. Already noted from New Caledonian waters (Laurin 1997; Bitner 2007a, 2009, 2010), *Thecidellina maxilla* is widely distributed in the SW Pacifc, from New Zealand to French Polynesia (Lee and Robinson 2003; Bitner 2007b, 2008, 2014; Logan 2007; MacFarlan et al. 2009).

## Summary

The brachiopods collected south of New Caledonia during the Terrasses cruise are represented by 15 species belonging to 13 genera. In the material from the Loyalty Ridge collected during the Exbodi cruise 19 species belonging to 16 genera have been identified. Fourteen species, i.e. *Novocrania* sp., *Basiliola beecheri*, *Basiliola lucida*, *Basiliolella grayi*, *Ebiscothyris bellonensis*, *Stenosarina crosnieri*, *Stenosarina globosa*, *Kanakythyris pachyrhynchos*, *Terebratulina pacifica*, *Fallax neocaledonensis*, *Septicollarina zezinae*, *Frenulina sanguinolenta*, *Annuloplatidia richeri* and *Campages mariae* are common to both collections. One species, *Xenobrochus africanus*, found in the Terrasses collection was not recognized in the Exbodi material, whereas *Neoancistrocrania norfolki*, *Xenobrochus indianensis*, *Eucalathis murrayi*, *Annuloplatidia curiosa*, and *Thecidellina maxilla* were found in the Exbodi cruise but not in the Terrasses cruise. *Eucalathis murrayi* was reported for the first time from the New Caledonian region.

Most species recognized here have a wide geographical distribution, being known either in the Western Pacific or in the Indo-West Pacific Province. Only four species, *Ebiscothyris bellonensis*, *Stenosarina globosa*, *Kanakythyris pachyrhynchos*, and *Annuloplatidia richeri* can be treated as endemic to the New Caledonian region.

With the new record of *Eucalathis murrayi* from New Caledonia, the total number of species recognized in this region is now 45 (d’Hondt 1987; Laurin 1997; Bitner 2007a, 2009, 2010, 2011, 2014; Bitner et al. 2008; Bitner and Cohen 2015), of which 8 are in common with New Zealand (compare Bitner 2010, 2014, and this study). The New Caledonian brachiopod fauna shows the greatest affinity with that from Fiji, sharing 11 of 22 species (Bitner 2006b, 2008).

## Appendix

**Table:** List of brachiopod-bearing stations and species per station.

Station	Location	Depth	Species
**Terrasses**			
**Loyalty Ridge**			
DW 3032	22°41’S, 168°58’E	760–820 m	*Basiliola beecheri*
DW 3039	23°57’S, 169°44’E	600–680 m	*Novocrania* sp.
DW 3040	23°58’S, 169°43’E	750–780 m	*Septicollarina zezinae* *Annuloplatidia richeri*
DW 3041	23°59’S, 169°44’E	800–840 m	*Basiliola beecheri* *Stenosarina crosnieri* *Fallax neocaledonensis*
DW 3042	23°56’S, 169°43’E	920–990 m	*Basiliola beecheri*
CP 3047	23°35’S, 169°37'E	266–267 m	*Basiliolella grayi* *Stenosarina crosnieri* *Fallax neocaledonensis*
**Norfolk Ridge**			
CP 3051	23°48'S, 168°17'E	410–530 m	*Stenosarina crosnieri* *Fallax neocaledonensis* *Campages mariae*
DW 3056	23°42'S, 168°01'E	250–330 m	*Basiliolella grayi*
DW 3059	23°40'S, 167°44'E	440–450 m	*Campages mariae*
DW 3060	23°39'S, 167°44'E	440–450 m	*Campages mariae*
DW 3062	23°22'S, 168°02'E	300–320 m	*Basiliolella grayi* *Frenulina sanguinolenta*
DW 3063	23°23'S, 168°00.4'E	430–480 m	*Terebratulina pacifica* *Fallax neocaledonensis* *Frenulina sanguinolenta*
CP 3065	23°21'S, 168°00'E	480–550 m	*Fallax neocaledonensis*
CP 3066	23°18'S, 167°59'E	650–790 m	*Stenosarina crosnieri* *Fallax neocaledonensis*
CP 3067	23°17'S, 167°58'E	800 m	*Stenosarina crosnieri*
CP 3068	23°16'S, 167°57'E	790 m	*Basiliola beecheri* *Stenosarina crosnieri* *Terebratulina pacifica* *Fallax neocaledonensis* *Campages mariae*
DW 3069	23°18'S, 168°05'E	300–320 m	*Basiliola lucida* *Stenosarina crosnieri* *Campages mariae*
CP 3070	23°18'S, 168°05'E	300–320 m	*Stenosarina crosnieri*
DW 3072	23°19'S, 168°16'E	180–220 m	*Terebratulina pacifica*
DW 3075	23°17'S, 168°14'E	270 m	*Basiliolella grayi*
DW 3076	23°14'S, 168°13'E	390–570 m	*Stenosarina globosa*
DW 3077	23°15'S, 168°14'E	420–540 m	*Stenosarina crosnieri* *Fallax neocaledonensis*
**SW Terrasses**			
DW 3078	22°29'S, 167°30'E	180–210 m	*Basiliolella grayi* *Campages mariae*
DW 3079	22°28'S, 167°29'E	300–420 m	*Basiliolella grayi*
DW 3082	22°29'S, 167°23'E	290 m	*Terebratulina pacifica*
DW 3083	22°27'S, 167°25'E	470–570 m	*Basiliola beecheri* *Terebratulina pacifica* *Fallax neocaledonensis*
DW 3086	22°15'S, 167°13'E	400 m	*Ebiscothyris bellonensis*
DW 3089	22°17'S, 167°12'E	390–410 m	*Stenosarina globosa*
DW 3090	22°16'S, 167°08'E	260 m	*Basiliolella grayi* *Campages mariae*
CP 3091	22°17'S, 167°09'E	260–270 m	*Fallax neocaledonensis*
DW 3093	22°06'S, 167°03'E	190–200 m	*Basiliolella grayi*
DW 3094	22°04'S, 167°03'E	250–300 m	*Basiliolella grayi*
**Norfolk Ridge**			
DW 3100	22°59'S, 168°23'E	260–320 m	*Basiliolella grayi* *Terebratulina pacifica*
DW 3102	22°59'S, 168°23'E	410–430 m	*Stenosarina globosa*
CP 3104	22°58'S, 168°21'E	410–470 m	*Kanakythyris pachyrhynchos*
DW 3106	23°02'S, 168°21'E	180–220 m	*Basiliolella grayi* *Campages mariae*
DW 3107	23°01'S, 168°23'E	380–440 m	*Kanakythyris pachyrhynchos* *Fallax neocaledonensis*
DW 3108	23°01'S, 168°239'E	370–440 m	*Kanakythyris pachyrhynchos*
DW 3109	23°01'S, 168°18'E	150–180 m	*Basiliolella grayi* *Kanakythyris pachyrhynchos* *Xenobrochus africanus*
DW 3110	23°02'S, 168°16'E	270–310 m	*Basiliolella grayi*
**Pine Island**			
DW 3120	22°44'S, 167°15'E	320–360 m	*Fallax neocaledonensis*
DW 3121	22°45'S, 167°13'E	380–400 m	*Fallax neocaledonensis*
DW 3122	22°47'S, 167°12'E	390–410 m	*Fallax neocaledonensis*
DW 3123	22°53'S, 167°13'E	420–450 m	*Fallax neocaledonensis*
DW 3124	22°54'S, 167°15'E	460 m	*Fallax neocaledonensis*
DW 3129	22°42'S, 167°15'E	110–130 m	*Frenulina sanguinolenta*

**Exbodi**			
**New Caledonia**			
DW 3784	22°13'S, 22°13'S	353–365 m	*Stenosarina crosnieri* *Fallax neocaledonensis*
DW 3785	22°15'S, 167°10'E	386–387 m	*Stenosarina globosa*
CP 3786	22°15'S, 167°13'E	406–442 m	*Stenosarina globosa*
DW 3787	22°13'S, 167°06'E	223–249 m	*Basiliolella grayi* *Campages mariae*
CP 3788	22°13'S, 167°07'E	264–273 m	*Basiliolella grayi* *Campages mariae*
CP 3789	22°11'S, 167°07'E	335–350 m	*Basiliolella grayi* *Campages mariae*
CP 3791	22°15'S, 167°19'E	750–863 m	*Ebiscothyris bellonensis*
CP 3792	22°18'S, 167°22'E	850–876 m	*Ebiscothyris bellonensis*
CP 3793	22°16'S, 167°23'E	951–1180 m	*Ebiscothyris bellonensis*
DW 3798	21°32'S, 166°21'E	478–480 m	*Basiliola beecheri*
CP 3834	22°06'S, 167°04'E	257–258 m	*Basiliolella grayi* *Campages mariae*
CP 3842	22°23'S, 167°22'E	756–769 m	*Ebiscothyris bellonensis*
CP 3843	22°22'S, 22°22'S	776–800 m	*Ebiscothyris bellonensis*
CP 3844	22°20'S, 167°22'E	815–970 m	*Ebiscothyris bellonensis*
DW 3845	22°30'S, 167°09'E	70–72 m	*Ebiscothyris bellonensis*
DW 3846	22°04'S, 168°38'E	396 m	*Basiliolella grayi*
CP 3848	22°03'S, 168°42'E	430–440 m	*Kanakythyris pachyrhynchos*
CP 3849	22°03'S, 168°41'E	360–560 m	*Fallax neocaledonensis*
CP 3851	22°19'S, 168°45'E	471–510 m	*Basiliola lucida* *Stenosarina crosnieri* *Kanakythyris pachyrhynchos* *Terebratulina pacifica* *Fallax neocaledonensis* *Campages mariae*
CP 3852	22°17'S, 168°43'E	582 m	*Stenosarina crosnieri* *Fallax neocaledonensis* *Campages mariae*
DW 3862	22°20'S, 169°01'E	400–520 m	*Neoancistrocrania norfolki* *Basiliola beecheri* *Annuloplatidia curiosa*
DW 3863	22°21'S, 168°59'E	540–660 m	*Frenulina sanguinolenta*
CP 3871	22°53'S, 169°25'E	580–780 m	*Basiliola beecheri*
DW 3872	22°54'S, 169°27'E	159–756 m	*Frenulina sanguinolenta* *Thecidellina maxilla*
DW 3880	22°22'S, 171°39'E	350 m	*Basiliolella grayi* *Terebratulina pacifica*
CP 3882	22°21'S, 171°40'E	288 - 361 m	*Basiliolella grayi*
CP 3883	22°21'S, 171°39'E	433–516 m	*Basiliolella grayi*
CP 3884	22°22'S, 171°38'E	521–567 m	*Basiliolella grayi* *Terebratulina pacifica*
CP 3885	22°23'S, 171°39'E	558–584 m	*Basiliolella grayi* *Terebratulina pacifica*
DW 3887	22°22'S, 171°42'E	257–298 m	*Basiliolella grayi*
DW 3889	22°25'S, 171°41'E	354 m	*Basiliolella grayi*
DW 3895	22°25'S, 171°40'E	380 m	*Stenosarina crosnieri*
DW 3896	22°19'S, 168°41'E	340–343 m	*Basiliola lucida* *Terebratulina pacifica*
CP 3898	22°18'S, 168°42'E	340–346 m	*Basiliola lucida* *Stenosarina crosnieri*
DW 3900	22°17'S, 168°41'E	355–357 m	*Basiliola lucida* *Stenosarina crosnieri* *Campages mariae*
DW 3902	19°53'S, 165°49'E	410 m	*Frenulina sanguinolenta*
DW 3903	19°52'S, 165°50'E	580 m	*Frenulina sanguinolenta* *Thecidellina maxilla*
DW 3905	19°50'S, 165°34'E	300 m	*Frenulina sanguinolenta* *Thecidellina maxilla*
DW 3906	19°50'S, 165°33'E	490–580 m	*Terebratulina pacifica* *Frenulina sanguinolenta* *Thecidellina maxilla*
DW 3907	19°50'S, 165°33'E	608–671 m	*Thecidellina maxilla*
CP 3911	19°50'S, 165°33'E	680–802 m	*Novocrania* sp. *Basiliola beecheri* *Eucalathis murrayi* *Septicollarina zezinae* *Annuloplatidia richeri*
DW 3913	19°45'S, 165°45'E	622 m	*Basiliola beecheri* *Annuloplatidia richeri*
DW 3916	19°52'S, 165°55'E	749–922 m	*Ebiscothyris bellonensis*
DW 3917	19°52'S, 165°55'E	753–951 m	*Stenosarina crosnieri*
DW 3918	19°52'S, 165°55'E	748–922 m	*Stenosarina crosnieri*
DW 3922	18°33'S, 164°21'E	525–560 m	*Frenulina sanguinolenta*
DW 3923	18°33'S, 164°20'E	580–703 m	*Frenulina sanguinolenta* *Thecidellina maxilla*
DW 3924	18°35'S, 164°23'E	730 m	*Frenulina sanguinolenta*
DW 3925	18°35'S, 164°19'E	388 m	*Neoancistrocrania norfolki* *Eucalathis murrayi* *Xenobrochus indianensis* *Frenulina sanguinolenta* *Thecidellina maxilla*
CP 3927	18°36'S, 164°20'E	381 m	*Frenulina sanguinolenta* *Campages mariae*
DW 3928	18°38'S, 164°20'E	362–402 m	*Frenulina sanguinolenta* *Thecidellina maxilla*
DW 3930	18°37'S, 164°26'E	448–464 m	*Frenulina sanguinolenta*
DW 3932	18°32'S, 164°21'E	500–1100 m	*Frenulina sanguinolenta* *Thecidellina maxilla*
DW 3933	18°32'S, 164°22'E	474 m	*Frenulina sanguinolenta* *Thecidellina maxilla*
DW 3939	18°36'S, 164°24'E	489–860 m	*Frenulina sanguinolenta*
DW 3940	18°36'S, 164°24'E	380–430 m	*Frenulina sanguinolenta*
